# *LZTR1* molecular genetic overlap with clinical implications for Noonan syndrome and schwannomatosis

**DOI:** 10.1186/s12920-022-01304-x

**Published:** 2022-07-15

**Authors:** Kirsten M. Farncombe, Emily Thain, Carolina Barnett-Tapia, Hamid Sadeghian, Raymond H. Kim

**Affiliations:** 1 Toronto General Hospital Research Institute, University Health Network, Toronto, ON Canada; 2 Bhalwani Familial Cancer Clinic, Princess Margaret Cancer Centre, University Health Network, Toronto, ON Canada; 3 Division of Neurology, Department of Medicine, University Health Network, University of Toronto, Toronto, ON Canada; 4 Ellen and Martin Prossermann Centre for Neuromuscular Diseases, Toronto General Hospital, University Health Network, Toronto, ON Canada; 5 Division of Medical Oncology and Hematology, Princess Margaret Cancer Centre, University Health Network, Sinai Health System, Toronto, ON Canada; 6 Division of Clinical and Metabolic Genetics, The Hospital for Sick Children, Ontario Institute for Cancer Research, Department of Medicine, University of Toronto, Toronto, ON Canada

**Keywords:** Noonan syndrome, Neurofibromas, Whole exome sequencing, *LZTR1*

## Abstract

**Background:**

Noonan syndrome (NS) is a genetic disorder characterized by developmental delays, typical facial gestalt and cardiovascular defects. *LZTR1* variants have been recently described in patients with NS and schwannomatosis, but the association, inheritance pattern and management strategy has not been fully elucidated. Here, we review the contribution of *LZTR1* in NS and describe a patient with a novel, likely pathogenic variant in *LZTR1*.

**Case presentation:**

A female patient was diagnosed with clinical NS at 8 months of age. She presented in adulthood when a brain and spine MRI identified plexiform neurofibromas; however, she did not meet the clinical criteria for Neurofibromatosis type 1. No pathogenic variants were identified through molecular genetic analysis of *NF1*, *SPRED1* and a multigene NS panel. Whole exome sequencing at age 23 identified a novel de novo likely pathogenic heterozygous variant in the *LZTR1* gene denoted as c.743G>A (p.Gly248Glu). Serial MRIs have shown stable imaging findings and the patient is being followed clinically by cardiology, neurology and medical genetics.

**Conclusions:**

We identified a novel mutation in the *LZTR1* gene, not previously reported in association with NS. This report provides additional evidence to support for the assessment of schwannomatosis in patients with *LZTR1*-NS and may have overlap with Neurofibromatosis type 1.

## Background

Noonan syndrome (NS) is a genetic multisystem disorder with a prevalence of 1 in 1000–2500 live births (1). This condition is characterised by varying developmental delays, distinctive facial features, congenital heart defects and short stature; clinical diagnosis is often based on these features (2). NS is part of a group of phenotypically similar developmental disorders (RASopathies), caused by germline variants in the genes within the RAS/MAPK signalling pathway (3).

There are multiple genes that cause NS, all linked to the RAS/MAPK signalling pathway (4, 5). Fifty percent of individuals with NS have a germline pathogenic variant (PV) in *PTPN11* (2). Other reported genes include *SOS1, RAF1, ROT1* and *KRAS*, occurring in 13%, 5%, 5% and < 5% of cases, respectively (2). PVs in other genes, including *BRAF, MAP2K1* and *NRAS*, have been identified in less than 1% of affected individuals (2). Another gene, *LZTR1*, has been recently identified as a causative gene in RASopathies (6); however, its precise role in the RAS/MAPK signalling pathway is less defined. Additionally, germline pathogenic variants in *LZTR1* have been identified in patients with schwannomatosis and is thought to be a distinct entity as schwannomas are not frequently seen in NS (7, 8). There is a paucity of cases to address this genetic and phenotypic heterogeneity in *LZTR1* carriers.

*LZTR1* (OMIM 600574), encoding leucine zipper-like transcription regulator 1 (LZTR1), was proposed as a tumor suppressor gene belonging to the BTB-Kelch superfamily (9). LZTR1 is a Golgi protein and belongs to the BTB-Kelch superfamily (7, 9), where it is reported to be involved in apoptosis (9) and acts as a substrate-specific adaptor for the Cullin-3 (Cul3) ubiquitin ligase (10, 11). Using a computational platform, *LZTR1* was identified as a tumor suppressor gene, with somatic mutations in this gene driving glioblastoma (10). Somatic mutations in *LZTR1* have also been associated with liver cancer (12).

Recent investigations have provided more insight into its potential role in the RAS/MAPK signalling pathway. *LZTR1* binds to the RAF1/SHOC2/PP1CB complex and promotes RAF1 Ser259 phosphorylation, leading to MAPK signalling pathway inactivation (13). Additional data reports that *LZTR1* enables the polyubiquitination and degradation of endogenous RAS, which ultimately inhibits RAS/MAPK signalling (14). Two other studies suggest that *LZTR1* mediates RAS ubiquitination and MAPK pathway activation, contributing to the development of human disease (15, 16). Examination of *LZTR1* variants associated with NS suggest this gene is functionally-linked to the RAS/MAPK pathway by negatively controlling RAS protein levels and MAPK signalling (17). In addition, a biological relationship has been proposed between LZTR1 and RIT1, whereby pathogenic mutations affecting RIT1 or LZTR1 leads to RIT1 accumulation and contributes to hyperactivation of MAPK signalling (18).

While the association of germline *LZTR1* variants with human disease is still being elucidated, germline loss-of-function mutations in *LZTR1* predispose to schwannomatosis (7, 19, 20) and NS (2). NS has wide genetic heterogeneity and clinical variability (21). Inheritance most frequently occurs in an autosomal dominant (AD) manner, however, PVs in *LZTR1* leading to NS can also be inherited in an autosomal recessive (AR) manner (2). Expression experiments suggest that *LZTR1* variants that cause AD NS may not be gain-of-function, whereas variants in patients with AR NS may have a loss-of-function effect (13). Loss-of-function mutations in biallelic *LZTR1* variants include splice site, frameshift, nonsense and missense modifications (17). Experiments assessing the functionality of missense *LZTR1* mutations suggests that schwannomatosis-associated *LZTR1* mutations act heterogeneously in modifying RAS-MAPK signalling, similar to variants causing dominant NS (17). The pleotropic function of LZTR1 may explain the multiple inheritance patterns (AR, AD) of *LZTR1*-associated NS. NS has been associated with Neurofibromatosis type 1 (NF1) [Neurofibromatosis-Noonan syndrome (NFNS)], a rare disorder where individuals present with phenotypic characteristics of these two autosomal dominant conditions (22). While there was an earlier debate about whether NFNS is a separate genetic condition, more recent reports illustrated clinical findings of both disorders, providing support that this is a new syndrome (23). NFNS is mainly caused due to mutations in *NF1* (24–28), however, there has occasionally been a *PTPN11* mutation reported in addition to the *NF1* gene mutation (29, 30). The involvement of other genes within the RAS-MAPK signalling pathway that cause NS have not yet been explored in NFNS (31). As such, NS should be considered part of the differential diagnosis in NF1 patients.

In this report, we describe a patient with clinically diagnosed NS who presented with sacral plexiform nerve sheath tumors, suggestive of neurofibromas or schwannomas. Whole exome sequencing (WES) revealed a de novo likely pathogenic heterozygous variant in the *LZTR1* gene. Here we demonstrate the clinical overlap of NS, schwannomatosis and NF1 and support the inclusion of *LZTR1* in gene panels for NS. In addition, we have reviewed the published literature on NS patients and variants in the *LZTR1* gene, noting zygosity, inheritance pattern and clinical characteristics, to inform the management of individuals with a heterozygous *LZTR1* mutation.

## Case presentation

### Case description

**Fig. 1:**
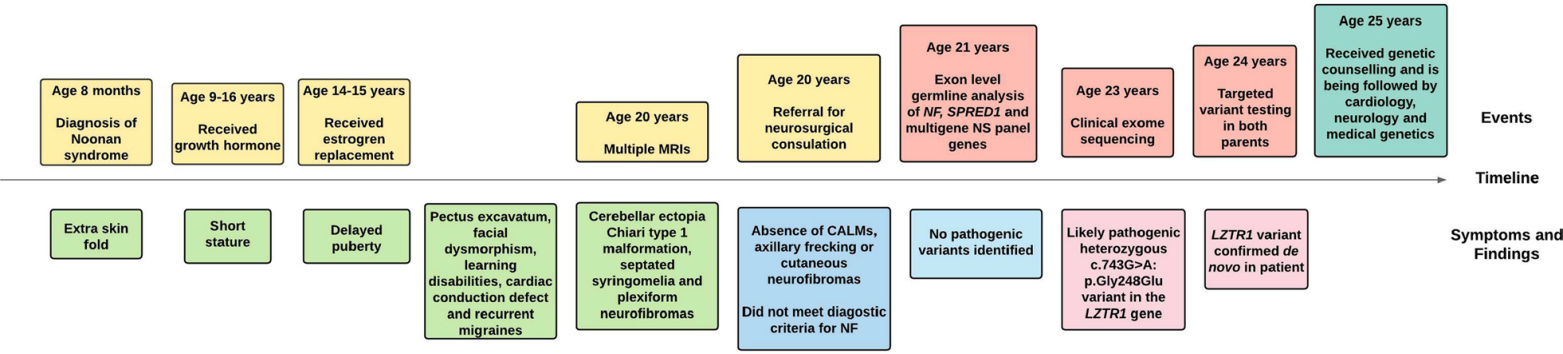
Timeline of the patient’s diagnosis, symptoms and treatment

A Caucasian female received a clinical diagnosis of NS at 8 months of age, prompted by an extra nuchal skin fold (Fig. 1). Facial features were consistent with NS (large eyes, low-set cupped ears and prominent lips) and she was also found to have congenital heart defects (subaortic ridge with mild left ventricular outflow tract obstruction, mitral valve prolapse with mild mitral regurgitation and tricuspid aortic valve with mild aortic insufficiency), recurrent migraines, pectus excavatum and a history of learning disability.

The patient received daily growth hormone (GH) injections as a child between ages 9–16 to address short stature. Her pre-growth hormone height was 118.6 cm (< 5%ile according to CDC growth charts and 50%ile in Noonan Syndrome growth charts) (32). No significant adverse effects were reported. Her height at adulthood (~ 17 years of age) was 158.4 cm, placing her in the 25%ile on the CDC Growth Charts and 90%ile on Noonan Syndrome growth charts.

Due to delayed puberty, the patient had a trial of transdermal estradiol at the age of 14. This was transitioned to Premarin with good gonadotropic effect, and she has been on oral contraceptives since 16 years of age.

At 20 years of age, a magnetic resonance imaging (MRI) brain and intracranial magnetic resonance angiography (MRA) was performed for chronic frontal headaches. MRA was normal. MRI of the brain and subsequent MRIs of the whole spine revealed a cerebellar ectopia related to a Chiari type 1 malformation and septated syringomelia extending from C1-T6 with no evidence of an underlying mass. There were multiple plexiform neurofibromas in the sacral spine, prompting a referral to neurosurgery for suspicion of NF1. At 23 years of age, a discontinuous expansile syrinx present from C6-T6 was noted, with enlargement of the dorsal root ganglia in the cervical spine.

**Fig. 2:**
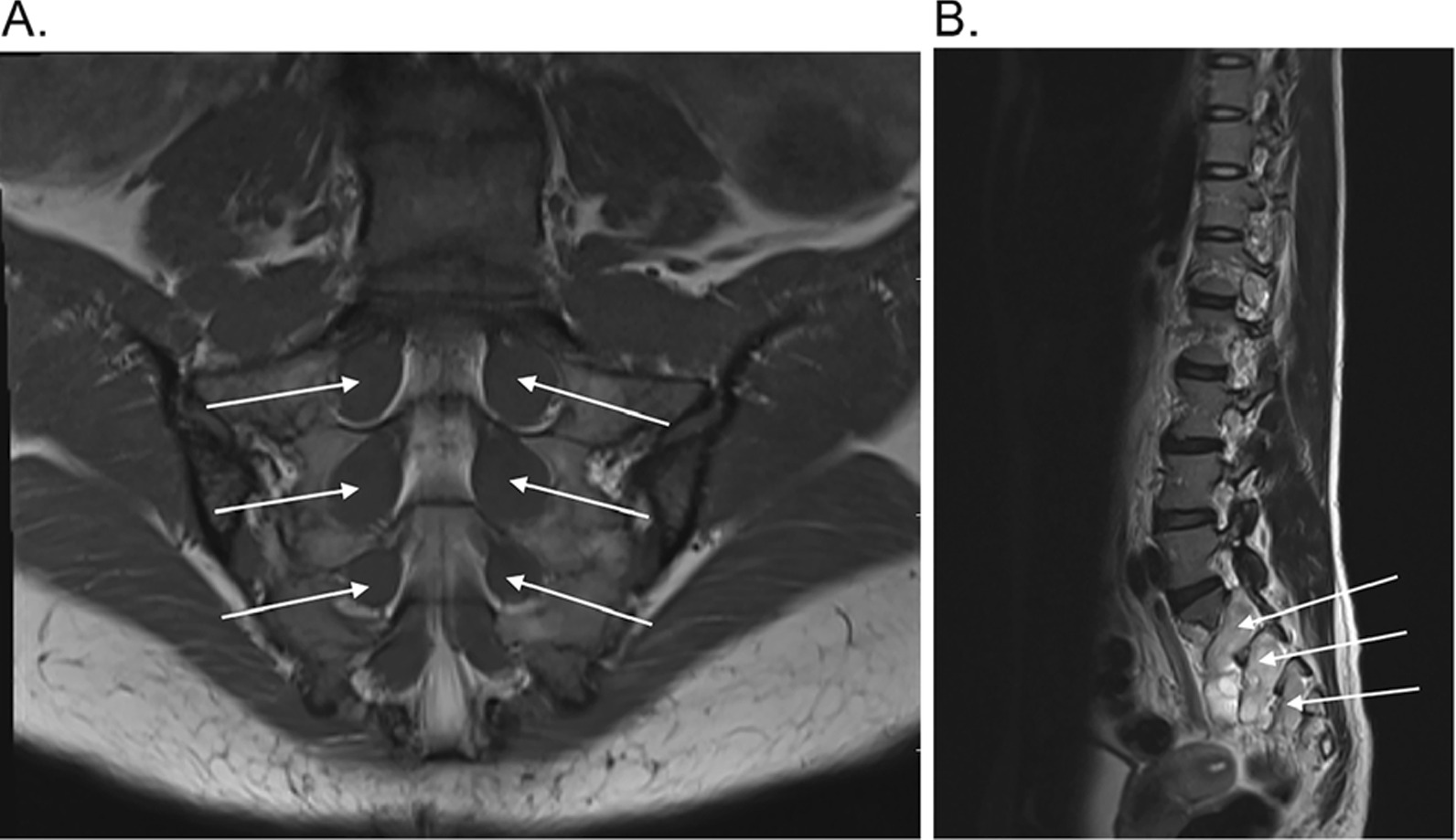
De-identified images of the patient’s plexiform neurofibromas. Panel **A** shows cornal T1 sequence, with multiple hypodense lesions arising from the sacral nerve roota, in keeping with neurofibromas (white arrows). Panel **B** shows sagittal T2 sequence, with neurofibromas seen as hyperintense lesions (white arrows)

The plexiform neurofibromas involved all nerve roots and caused marked expansion of the sacral foramina. As a biopsy was unlikely to change management, these lesions were not biopsied and thus imaging findings were not pathologically confirmed (Fig. 2A–B). Serial MRIs have shown stable imaging findings. Upon clinical examination, the patient did not present with café-au-lait macules (CALMs), skinfold freckling, or cutaneous or subcutaneous neurofibromas. Due to the initial question of NF1, genetic testing was conducted in a step wise fashion to establish a molecular genetic diagnosis.

### Next generation sequencing

Exon-level germline analysis of *NF1* [NCBI RefSeq NM_000267.3], *SPRED1* [NM_152594.2] and a multigene NS panel (*BRAF* [NM_004333.4], *CBL* [NM_005188.3], *HRAS* [NM_005343.2], *KRAS* [NM_004985.3], *MAP2K1* [NM_002755.3], *MAP2K2* [NM_030662.3], *NRAS* [NM_002524.4], *PTPN11* [NM_002834.3], *RAF1* [NM_002880.3], *RIT1* [NM_006912.5], *SHOC2* [NM_007373.3], *SOS1* [NM_005633.3]) was completed on DNA extracted from blood leukocytes at The Hospital for Sick Children Genome Diagnostics Laboratory (Toronto, ON). Next Generation Sequencing (NGS) was performed using a targeted Agilent SureSelect custom capture followed by paired-end sequencing using the Illumina sequencing platform. Variant calls were generated using Genomic Analysis Tool Kit (GATK) after read alignment with the Burrows-Wheeler Aligner (BWA). Genome build NCBI37/hg19 with decoy and data analysis software SK High Coverage Clinical Pipeline was used. Multiplex ligation-dependent probe amplification (MLPA) was used to test gene dosage.

Clinical exome sequencing analysis (NGS with copy number variant [CNV]) was completed by the GeneDx Molecular Laboratory (Gaithersburg, USA) on DNA obtained via a buccal swab. The enriched targets were simultaneously sequenced with paired-end reads on an Illumina platform and genome build GRCh37/UCSC hg19. Using a custom-developed analysis tool (XomeAnalyzer), data were filtered and analysed to identify sequence variants and most deletions and duplications involving three or more coding exons (33). Sequence and CNVs are reported according to the Human Genome Variation Society (HGVS) or International System for Human Cytogenetic Nomenclature (ISCN) guidelines, respectively. Reportable PVs, likely PVs and variants of uncertain significance were reported as per AMP/ACMG guidelines (34). Secondary findings were examined in the coding regions of the gene list provided by ACMG SF v2.0 (September 2016) (35).

### Molecular genetic approach

Exon-level germline genetic testing of the *NF1, SPRED1,* and a multigene NS panel that did not include the *LZTR1* gene did not reveal any PVs in the genes tested. A single nucleotide polymorphism (SNP) microarray at the same laboratory was in-keeping with a normal female, arr(1-22,X)×2. As schwannomas and neurofibromas are not typically reported in patients with NS, the molecular genetic approach in patients with nerve sheath tumors often begins with NF1. For this patient, multi-gene panel testing for NS, NF1 and Legius syndrome were negative and a negative SNP chromosomal microarray. Of note, due to the emerging literature for *LZTR1* in NS, *LZTR1* was not yet included in the laboratory’s NS gene panel.

Clinical exome sequencing analysis identified a likely pathogenic heterozygous c.743G>A (p.Gly248Glu) in exon 8 of the *LZTR1* gene [NM_006767.3]. Variants at this residue are normally associated with an autosomal dominant disorder. These findings are consistent with the patient’s reported clinical features. No reportable secondary findings were identified and was in accordance to the reporting structure recommended by the American College of Medical Genetics (ACMG) (35, 36).

The p.Gly248Glu variant identified in this study is located in the Kelch domain of *LZTR1*; heterozygous missense mutations in this region have been seen in patients with a clinical diagnosis of NS (6), NS patients with heterozygous PVs in *LZTR1* (37), as well as in NS patients with a bleeding phenotype (8, 38). This variant has previously been identified in a patient with fetal pleural effusion and NS (39) and has not been observed in larger population cohorts (40).

Parental targeted variant testing confirmed this was a de novo variant in the patient. Her parents have no features of NS, supporting the causality of this variant. The patient and her family have undergone genetic counselling regarding implications of the results for her, her family and any future children. She is being followed clinically by cardiology, neurology and medical genetics.

## Discussion and conclusions

Monoallelic and biallelic *LZTR1* variants in NS are not well described, however, previous studies have reported germline variants causative for NS (Table 1) in either an autosomal recessive or autosomal dominant manner (6, 8, 13, 37, 38, 41–47). Of note, a different missense mutation at the same protein residue as our patient (p.Gly248Arg) was reported as pathogenic or likely pathogenic in other individuals with NS (6, 13, 38, 47), with the same designations on ClinVar. In silico analysis of variants (p.Ala116Val, p.Arg284Cys, p.Arg688Cys, p.Gly248Arg, p.His287Tyr, p.Pro520Leu, p.Ser247Asn, p.Ser122Leu, p.Tyr119Cys and p.Val456Gly) found in the *LZTR1* gene support a deleterious effect (6, 7, 42).

**Table 1:** Previous published reports of Noonan syndrome and *LZTR1* variants

Variant(s)	Zygosity	Mode of inheritance	Clinical diagnosis/Features	PMID
c.742G>A (p.Gly248Arg)	Heterozygous	Maternal	**Proband**: typical facial features, short/webbed neck, pectus deformity, pulmonary valve stenosis/atrial septal defect, ophthalmological abnormality (prominent corneal nerves), lacrimal duct obstruction **Mother**: typical facial features, short/webbed neck, pectus deformity, mitral valve prolapse, height -2.5 SDS **Grandfather**: typical facial features, short/webbed neck, pectus deformity, mitral valve prolapse, abnormal hemostasis (Factor XI deficiency), height -2.9 SDS	25795793
c.850C>T (p.Arg284Cys)	Heterozygous	Maternal	**Proband**: typical facial features, abnormal hemostasis (prolonged ATTP) **Mother**: typical facial features, nevi **Sibling 1**: typical facial features, ectodermal findings (curly hair, sparse eyebrows, hyperkeratosis pilaris), nevi **Sibling 2**: typical facial features, short/webbed neck, ectodermal findings (curly hair), hemangioma **Half Sibling 1**: typical facial features **Half Sibling 2**: typical facial features, height -3.8 SDS	25795793
c.859C>T (p.His287Tyr)	Heterozygous	De novo	**Proband**: typical facial features, pulmonary valve stenosis/atrial septal defect, cryptorchidism, abnormal hemostasis (prolonged ATTP), ophthalmological abnormality (prominent corneal nerves), developmental delay, learning disability	25795793
c.356A>G (p.Tyr119Cys)	Heterozygous	De novo	**Proband**: typical facial features, left ventricular hypertrophy, lymphedema, varicose veins	25795793
c.740C>A (p.Ser247Asn)	Heterozygous	Maternal	**Proband**: typical facial features, short/webbed neck, pectus deformity, mitral valve insufficiency, ectodermal findings (curly hair), developmental delay, learning disability, **Mother**: typical facial features, short/webbed neck, aorta coarctation, hyperopia, tumors (neurinomas of right hand and forearm, lipoma of thorax = schwannomas)	25795793
c.881G>T (p.Arg294Leu)/ c.2212C>T (p.Gln738*)	Compound heterozygous	Maternal/paternal	**Proband**: typical NS facial features, pectus excavatum, short stature treated with growth hormone, growth hormone deficiency, thickening of the left side of the optic chiasm suggestive of glioma, Senning correction surgery for transposition of the great vessels, pulmonary stenosis, interventricular and interatrial communication	29959388
c.509G>C (p.Arg170Pro)/ c.2374T>G (p.Cys792Gly)	Compound heterozygous	Maternal/paternal Autosomal recessive	**Proband**: NS clinical phenotype, short stature, left ventricular outflow tract obstruction, atrial septal defect	30732632
c.850C>T (p.Arg284Cys)	Heterozygous	Maternal Dominant	**Proband**: Typical NS dysmorphism, Charcot-Marie-Tooth syndrome, manual dyspraxia and distal muscular weakness, short statue treated with growth hormone, scoliosis and lumbar scoliosis, partial complex seizures leading to identification of a right fronto-temporo-insular tumor, severe kyphoscoliosis with a gibbus, pectus excavatum, generalized amyotrophy, mild defect in factor XI, grade IV gliomablastoma	30664951
c.1149+1G>T	Heterozygous	Maternal Autosomal dominant	**Proband**: typical NS appearance, short stature, delayed psychomotor development, frequent premature ventricular beats, hemivertebra deformity, scoliosis, refractive errors, growth hormone deficiency, pectus excavatum, café au lait spots, mild hypertrichosis **Sibling**: typical NS appearance, pectus carinatum, short stature **Mother**: mild typical NS appearance	33407364
c.1084C>T (p.Arg362*)/ c.1149+1G>T	Compound heterozygous	Maternal/paternal Autosomal recessive	**Proband**: severe hypertrophic cardiomyopathy, mild pulmonary valve stenosis, characteristic NS facies, broad QRS complexes, right bundle branch block, left axis deviation, striking negative pattern in the left precordial leads	31182298
c.2070-2A>G/ c.1735G>A (p.Val579Met)	Compound heterozygous	Paternal/maternal Autosomal recessive	**Proband**: severe hypertrophic cardiomyopathy without obstruction, left axis deviation, negative pattern in the left precordial leads, severe feeding problems	31182298
c.355T>C (p.Tyr119His)	Not provided	De novo	**Proband**: typical craniofacial dysmorphology, pulmonary valve stenosis/branch pulmonary arterystenosis, slight asymmetric hypertrophy of inteventricular sept, café au lait spots, nevi or lentigines, permanence of fetal finger and toepads, narrow palate	32514133
c.1430C > T (p.Ala477Val)/ three *LRP1* variants	Heterozygous	Paternal/maternal	**Proband**: delayed development, height -4.98 SD, typical craniofacial dysmorphology, broad thorax with wide-spaced nipples, cubitus valgus, clinobrachydactyly, cryptorchidism, GH deficiency, previous epilepsy (rolandic type, absences), thoracolumbar scoliosis, generalized hirsutism	32514133
c.347C > T (p.Ala116Val)	Heterozygous	De novo	**Proband**: typical facial dysmorphism, height -4.3 SD, short webbed neck with low posterior hairline, pectus deformity, heart murmur, hypertrophic cardiomyopathy, cryptorchidism, ostium secundum atrial septal defect, mitral anomaly, ectodermal findings, sparse eyebrows, ulerythema ophriogenes, developmental delay	https://doi.org/10.4172/0974-8369.1000414
c.628C > T (p.Arg210*)/c.2220-17C>A (p.Tyr741Hisfs*89)	Compound heterozygous	Paternal/maternal Autosomal recessive	**Sibling 1**: typical facial dysmorphism, broad/short neck, low posterior hairline, pectus carinatum or excavatum, congenital heart defect or valvular disease **Sibling 2**: prenatal hydrops, nuchal transl or cardiac findings, typical facial dysmorphism, broad/short neck, low posterior hairline, pectus carinatum or excavatum, congenital heart defect or valvular disease, curly hair, developmental delay **Sibling 3**: typical facial dysmorphism, broad/short neck, low posterior hairline, pectus carinatum or excavatum, wide-spaced nipples/broad chest, leukemia **Sibling 4**: prenatal hydrops, nuchal transl or cardiac findings, typical facial dysmorphism, broad/short neck, low posterior hairline, pectus carinatum or excavatum, wide-spaced nipples/broad chest, congenital heart defect or valvular disease, height < 3rd centile **Several individuals** in this family had suggestive schwannomas	29469822
c.2178C>A (p.Tyr726*)/ c.1943-256C > T	Heterozygous	Paternal/maternal Autosomal recessive	**Sibling 1**: prenatal hydrops, nuchal transl or cardiac findings, typical facial dysmorphism, broad/short neck, low posterior hairline, wide-spaced nipples/broad chest, pectus carinatum or excavatum, cardiomyopathy, congenital heart defect or valvular disease, cryptorchidism, developmental delay/intellectual disability, height < 3^rd^ centile **Sibling 2**: prenatal hydrops, nuchal transl or cardiac findings, typical facial dysmorphism, broad/short neck, cardiomyopathy, congenital heart defect or valvular disease, developmental delay/intellectual disability, height 3^rd^ centile	29469822
c.1943-256C > T; *70G>A/ c.1943-256C > T; *70G>A	Homozygous	Paternal/maternal Autosomal recessive	**Sibling 1**: prenatal hydrops, nuchal transl or cardiac findings, depressed or wide bridge, low set ears, broad/short neck, low posterior hairline, wide-spaced nipples/broad chest, cardiomyopathy, height < 3^rd^ centile **Sibling 2**: prenatal hydrops, nuchal transl or cardiac findings, ptosis, downslanted palpebral fissures, low set ears, cardiomyopathy, congenital heart defect or valvular disease, height < 5-10^th^ centile	29469822
c.1687G>C (p.Glu563Gln)	Homozygous	Paternal/maternal Autosomal recessive	**Sibling 1**: typical facial dysmorphism, broad/short neck, low posterior hairline, pectus carinatum or excavatum, cardiomyopathy, congenital heart defect or valvular disease, developmental delay/intellectual disability **Sibling 2**: prenatal hydrops, nuchal transl or cardiac findings, broad/short neck, cardiomyopathy, congenital heart defect or valvular disease, cryptorchidism	29469822
c.2407-2A>G/ c.2090G>A (p.Arg697Gln)	Compound heterozygous	Paternal/maternal Autosomal recessive	**Sibling 1**: prenatal hydrops, nuchal transl or cardiac findings, typical facial dysmorphism, broad/short neck, wide-spaced nipples/broad chest, curly hair, cardiomyopathy, congenital heart defect or valvular disease, height 3^rd^ centile **Sibling 2 (twins)**: prenatal hydrops, nuchal transl or cardiac findings, broad/short neck, wide-spaced nipples/broad chest, congenital heart defect or valvular disease	29469822
c.27delG (p.Gln10fs*15)/ c.1149+1G>A	Compound heterozygous	Paternal/maternal Autosomal recessive	**Proband**: typical facial dysmorphism, broad/short neck, wide-spaced nipples/broad chest, height < 5^th^ centile	29469822
c.361C > G (p.His121Asp)/ c.2264G>A (p.Arg755Gln)	Compound heterozygous	Paternal/maternal Autosomal recessive	**Proband**: prenatal hydrops, nuchal transl or cardiac findings, typical facial dysmorphism, broad/short neck, low posterior hairline, wide-spaced nipples/broad chest, pectus carinatum or excavatum, curly hair, cardiomyopathy, congenital heart defect or valvular disease, developmental delay/intellectual disability, height < 3^rd^ centile	29469822
c.508C > T (p.Arg170Trp); c.614T>C (p.Ile205Thr)/ c.508C > T (p.Arg170Trp); c.614T>C (p.Ile205Thr)	Compound homozygous	Paternal/maternal Autosomal recessive	**Proband**: typical facial dysmorphism, broad/short neck, curly hair, cardiomyopathy, congenital heart defect or valvular disease, cryptorchidism developmental delay/intellectual disability, height < 3^rd^ centile	29469822
c.650A > C (p.Glu217Ala)/ c.650A > C (p.Glu217Ala)	Homozygous	Paternal/maternal Autosomal recessive	**Proband**: typical facial dysmorphism, broad/short neck, cardiomyopathy, congenital heart defect or valvular disease, developmental delay/intellectual disability, height 3^rd^ centile	29469822
c.2062C > G (p.Arg688Gly)/ c.1943-256C > T	Heterozygous	Paternal/maternal Autosomal recessive	**Sibling 1**: prenatal hydrops, nuchal transl or cardiac findings, typical facial dysmorphism, broad/short neck, wide-spaced nipples/broad chest, cardiomyopathy, developmental delay/intellectual disability, height < 3^rd^ centile	29469822
c.2325+1G>A/ c.1943-256C > T	Not provided	Paternal/maternal Autosomal recessive	**Sibling 1**: prenatal hydrops, nuchal transl or cardiac findings, typical facial dysmorphism, broad/short neck, wide-spaced nipples/broad chest, pectus carinatum or excavatum, cardiomyopathy, congenital heart defect or valvular disease	29469822
c.2462T>C (p.Ile821Thr)/ c.2462T>C (p.Ile821Thr)	Homozygous	Paternal/maternal Autosomal recessive	**Sibling 1**: typical facial dysmorphism, low posterior hairline, wide-spaced nipples/broad chest, pectus carinatum or excavatum, curly hair, cardiomyopathy, congenital heart defect or valvular disease, developmental delay/intellectual disability **Sibling 2**: low set ears, post angulated ears, midface retrusion, low posterior hairline, wide-spaced nipples/broad chest, pectus carinatum or excavatum, cardiomyopathy, congenital heart defect or valvular disease, developmental delay/intellectual disability, height < 3^rd^ centile	29469822
c.406T>C (p.Tyr136His)	Heterozygous	De novo Autosomal dominant	**Proband**: abnormalities of the abdomen, atrioventrical valves, heart values and spatial orientation of the cardiac segments, coarctation of the aorta, heptamegaly, large for gestational age, malrotation of small bowel, malformation of the heart and great vessels, height 2-9^th^ centile, mild typical facial dysmorphism	30859559
c.434A > T (p.Asn145Ile)	Heterozygous	Maternal Autosomal dominant	**Proband**: short stature, characteristic NS facial features, mild pulmonary stenosis, mild learning difficulties, mild right bundle branch block with mild left ventricular dilation with a normal pulmonary valve, low posterior hairline, widely spaced nipples, mild pectus excavatum, café-au-lait macule, platelet dysfunction disorder **Mother**: mild clotting disorder, type 1 von Willebrand’s disease, mild learning difficulties, mild NS-like facial features **Cousin**: Cerebral Palsy, characteristic NS facial features, periventricular leukomalacia, global developmental delay, abnormal gait with increased limb tone, moderate learning difficulties, behavioural issues within the autistic spectrum disorders **Aunt**: childhood growth delay, delayed puberty, hypothyroidism, underweight **Half Aunt**: heart murmur, mitral valve prolapse, small spina bifida, subtle facial features of NS	30859559
c.290G > T (p.Arg97Leu)	Not provided	De novo Autosomal dominant	**Proband**: prenatal cardiac findings (absent ductus venosus), atrial septal defect, typical NS facial features, downslanted palpebral fissures, epicanthus, wide spaced nipples/broad chest, curly hair hypertrophic cardiomyopathy, short stature, ventricular septal defect, foot abnormalities, delayed walking, scoliosis, impaired clotting, respiratory problems	30859559
c.407A>G (p.Tyr136Cys)	Not provided	De novo Autosomal dominant	**Proband**: 2–3 toe syndactyly, typical NS facial features, barrel-shaped chest, cryptorchidism, delayed speech and language development, depressed nasal bridge, wide spaced nipples/broad chest, curly hair, generalised hypotonia, low-set posteriorly rotated ears, motor delay, unilateral ptosis, wide intermammillary distance, GH deficiency, mild pulmonary valve stenosis, short stature	30859559
c.731C > G (p.Ser244Cys)	Not provided	De novo Autosomal dominant	**Proband**: café-au-lait spots, hypermetropia, typical NS facial features, low-set posteriorly rotated ears, wide spaced nipples/broad chest, pectus carinatum, webbed neck, short stature, single transverse palmar crease, strabismus, webbed neck	30859559
c.742G>A (p.Gly248Arg)	Not provided	De novo Autosomal dominant	**Proband**: prenatal hydrops, depressed nasal bridge, epicanthus, microcephaly, preauricular pit, prominent metopic ridge, severe global developmental delay, perintal asphyxia, seizures, underweight, height 1^st^ centile, marked micrognathia and feeding problems, facial features now atypical for NS, cubitus valgus, pectus excavatum, renal abnormalities, valvular heart disease	30859559
c.1591G>A (p.Asp531Asn)/ c.628C > T (p.Arg210*)	Compound heterozygous	Autosomal recessive	**Proband**: autistic behaviour, global developmental delay, hypertrophic cardiomyopathy, long palpebral fissure, mitral valve prolapse, pes plantus, prominent fingertip pads, tonic–clonic seizures, facial features suggestive of Kabuki syndrome, height 3^rd^ centile, cryptorchidism	30859559
c.1149+1G > T/ c.2062C > T (p.Arg688Cys)	Compound heterozygous	Autosomal recessive	**Proband**: Bilateral ptosis, typical NS facial features, blue irides, downslanted palpebral fissures, hyperacusis, hypertelorism, joint hypermobility, square thumb, low-set posteriorly rotated ears, pectus carinatum, broad/short neck, wide spaced nipples/broad chest, proportionate short stature, mild developmental delay, delayed speech and language development	30859559
c.628C > T (p.Arg210*)/ c.1735G>A (p.Val579Met)	Compound heterozygous	Autosomal recessive	**Proband**: rare biallelic variants in *NEB* lead to a “blended” phenotype, nuchal translucency abnormalities, typical NS facial features, blifid uvula, bilaterial ptosis, downslanted palpebral fissures, generalised joint laxity, hearing impairment, high palate, hypertelorism, long face, macrodontia, myopathy, pectus excavatum, pointed chin, renal duplication, retrognathia, mitral valve regurgitation, duplex kidney, mild developmental delay, delayed speech and language development, delayed walking, hypertonia, feeding problems, easy bruising	30859559
c.1311G>A (p.Trp437*)/ c.-38T>A	Compound heterozygous	Suspected autosomal recessive	**Proband**: typical NS facial features, prenatal hydrops, nuchal translucency abnormalities, broad/short neck, webbed neck, low posterior hairline, wide spaced nipples/broad chest, left ventricle hypertrophy, foot abnormalities, delayed walking, hypotonia, joint laxity/ hypermobility **Sibling**: affected, died at birth	30859559
c.1407G>A (p.Trp469*)/ c.2246A>G (p.Tyr749Cys)	Compound heterozygous	Suspected autosomal recessive	**Proband**: typical NS facial features, severe hypertrophic cardiomyopathy, broad/short neck, webbed neck, low posterior hairline, wide spaced nipples/broad chest, pes planus, mild developmental delay, impaired vision	30859559
c.1382C>A (p.Ala461Asp)/ c.1385T>C (p.Ile462Thr)	Compound heterozygous	Maternal/paternal Suspected autosomal recessive	**Proband**: typical NS facial features, prenatal cardiomyopathy, broad/short neck, webbed neck, low posterior hairline, cubitus valgus, curly hair, cardiac hypertrophy, pulmonic stenosis, moderate developmental delay, delayed speech and language development	30859559
c.848G> A (p.Arg283Gln)	Heterozygous	De novo	**Proband**: dysmorphic features, short stature, short neck, webbed neck, scoliosis, hyperelastic skin, hyperkeratosis, wrinkled palms and soles, café au lait spots, atrial septal defect, ventricular septal defect, pulmonary stenosis, patent ductus arteriosus, severe intellectual disability	30368668
c.742G>A (p.Gly248Arg)	Heterozygous	Maternal Suspected autosomal dominant	**Proband**: Noonan-like syndrome, nuchal translucency, hypertelorism, ptosis, low-set ears, highly arched palate, anomalous origin of coronary artery, concealed penis **Mother:** ventricular septal defect, mild hypertelorism, downslanting palpebral fissures	30368668
c.2102C>A (p.Pro701His)/ c.2069+2T>C	Compound heterozygous	Paternal/maternal Suspected autosomal recessive	**Proband**: hypertelorism, low-set ears, sparse eyebrows, short stature, short neck, scoliosis, pectus excavatum, hyperpigmentation, hypertropic cardiomyopathy, mild intellectual disability, squint, amblyopia, 5^th^ brachymetapody	30368668
c.428A>G (p.Asn143Ser)	Heterozygous	Paternal	**Proband**: relative macrocephaly, typical NS facial features, short stature, pectus carinatum, hyperkeratosis, wrinkled palms and soles, hyperpigmentation, naevus, hypertropic cardiomyopathy, cryptorchidism, intellectual disability, puffy palms	30368668
c.606_650del (p.Met202fs)	Heterozygous	Paternal	**Proband**: pleural effusion, relative macrocephaly, typical NS facial features, short stature, short neck, webbing of neck, hypertropic cardiomyopathy, atrial septal defect, pulmonary stenosis, arrhythmia	30368668
c.756_758del (p.Asn253del)	Heterozygous	De novo	**Proband**: relative macrocephaly, typical NS facial features, short stature, short neck, webbing of neck, cubitus valgus, pectus carinatum, curly hair, hyperkeratosis, wrinkled palms and soles, hyperpigmentation, hypertropic cardiomyopathy, mild intellectual disability, visual field contraction, optic atrophy	30368668
c.1660G>C (p.Ala554Pro)	Heterozygous	Paternal	**Proband**: relative macrocephaly, typical NS facial features, short neck, webbing of neck, cubitus valgus, curly hair, hyperelastic skin, hyperkeratosis, wrinkled palms and soles, hyperpigmentation, naevus, hypertropic cardiomyopathy, atrial septal defect, intellectual disability	30368668
c.742G>A (p.Gly248Arg)	Heterozygous	De novo	**Proband**: cryptorchidism, choroid plexus cyst, mild pulmonary supravalvular stenosis, typical NS facial features, curly hair	31533111
c.2074T>A (p.Phe692Leu)	Homozygous	Maternal/paternal	**Proband**: short stature, mild pulmonary supravalvular stenosis, Von Willebrand disease, cryptorchidism, orchidopexy, typical NS facial features, webbed neck	31533111
c.730T>C (p.Ser244Pro)	Heterozygous	Maternal	**Proband**: Chiari malformation type I, mild dorsal syringomyelia, hemangioma in the posterior cervical region, mastocytosis, high arched palate, crowded teeth, typical NS facial features, height lower end of the normal range, webbed neck **Mother**: mild facial features, curly hair, height lower end of the normal range, pectum excavatum, webbed neck	31533111

Our patient’s phenotype is unique, as not many patients with NS present with plexiform nerve sheath tumors. These are typically benign tumors, with neurofibromas characteristic of NF1 (48) and schwannomas often occurring in patients with NF2 or schwannomatosis (49). This case highlights one of the main issues in identifying NS patients—there is a degree of phenotypic overlap and uncertainty between other similar conditions; therefore, the focus of genetic testing may be on this rare manifestation with a misdiagnosis of NF1, NF2 or schwannomatosis, rather than NS. A recent study performed an extensive literature review to estimate the number of conditions that may mimic NF1, as well as examining data from 40 pediatric patients with NF1-like syndromes (50). Phenotypic overlap, particularly with skin manifestations or tissue overgrowth, was observed between NF1, NF1-like syndromes, the RASopathies and other disorders associated with higher tumor development, including phosphatase and tensin homolog (PTEN) hamartoma tumor syndromes, constitutional mismatch repair deficiency (CMMRD) syndromes, chromosomal abnormalities and multiple endocrine neoplasia (MEN) syndromes (50). Furthermore, genotypic overlap can also be seen between NF1-like syndrome and other disorders, including NS (50).

Besides our patient, there have been a few reports of neurofibromas/schwannomas in patients with a RASopathy phenotype. One patient presented with an overgrowth of peripheral nerve sheaths, suggestive of a schwannoma or neurofibroma (51). The final diagnosis was multiple diffuse schwannomas; although this is suggestive of NF2 or schwannomatosis, a germline *KRAS* variant (p.Lys5Glu) was identified (51). In one family with unaffected parents and four affected children with NS, a c.2220-17C>A (p.Tyr741Hisfs*89) maternally-inherited splice variant was found; several members of this family had suggestive signs of schwannomas on MRI analysis (37). Another individual with a c.740C>A (p.Ser247Asn) variant in *LZTR1* developed multiple schwannomas in the right arm, however, no material from the schwannomas was available for molecular testing (6). This individual was the mother of a patient with NS (6).

Carriers of *LZTR1* have phenotypic heterogeneity and management of *LZTR1*-NS patients has not been well described. Guidelines regarding the management of individuals with NS varies and is based on the clinical manifestations. Some common treatments include: treatment for cardiovascular anomalies, early intervention programs for developmental disabilities, treatment for serious bleeding conditions, GH treatment for short stature and further monitoring for abnormalities (2). In comparison, germline or mosaic mutations in the *LZTR1* (7, 19, 20) and the *SMARCB1* (52) genes have been associated with schwannomatosis, although the link between *LZTR1* schwannomatosis and the development of other tumors has not been clearly defined. Current clinical management for schwannomatosis recommends that individuals have a baseline brain and spine MRI in late childhood/early adulthood to monitor the disease and management in adulthood should be performed by a neurologist or neurofibromatosis specialist to manage pain (53). Whole body MRI and increased surveillance can be considered if the patient is symptomatic (53). The guidelines on how to manage *LZTR1*-NS and *LZTR1*-schwannamatosis patients are not clear; therefore, a reasonable approach would be management of patients under both NS and schwannomatosis guidelines. Clarification of the genetic etiology of NS should be pursued early in the course of NS management as this may have implications on the use of GH therapy in a patient with *LZTR1*-NS. This includes consideration of tumor development and cardiac abnormalities, as individuals with NS are at increased risk, and there have been reports of tumors (54–57) and adverse cardiac reactions (58) following GH therapy.

Our proband’s likely PV in the *LZTR1* gene was not detected on the original *NF1, SPRED1* and multigene NS panel. Eventually, clinical exome sequencing analysis was able to identify the causative gene, which was later confirmed to be absent in her clinically unaffected parents. In 2018, the Clinical Genome Resource (ClinGen) RASopathy expert panel assessed 19 genes, including *LZTR1*, and found a strong association between *LZTR1* and AD NS (59). As of 2020, the RASopathy expert panel definitively associates *LZTR1* with AD NS and has stated there is strong evidence between *LZTR1* and AR NS. Since then, *LZTR1* has been a common new addition to many commercial and academic and laboratory NS panels. Based on our findings, inclusion of additional NS genes to schwannomatosis panels could also be considered. Due to the high de novo rate of variants causing NF1, NS and schwannomatosis, an alterative approach may be trio whole exome sequencing to aid in the interpretation of variants.

Based on previous reports and our findings, individuals with NS and a monoallelic or biallelic germline *LZTR1* mutation may be at an increased risk of developing schwannomas and may meet the diagnostic criteria for schwannomatosis. Additional case reports of NS and variants in *LZTR1*, as well as functional studies showing the involvement of *LZTR1* in the RAS/MAPK pathway would provide support for the creation of management guidelines for *LZTR1*-NS individuals. Due to the difficulties with overlapping phenotypes and genotypes, we believe that all NF1-like syndromes should be assessed in young individuals when identifying a causative gene and disorder. As clinical guidelines and gene panels change, clinicians should perform annual assessments of these patients to re-evaluate their original diagnosis and management. Due to the clinical implications for this patient presenting with schwannomas and NS, our patient will be followed as per NS and schwannomatosis surveillance guidelines to screen for nervous system tumors and monitor for tumor development.

## Data Availability

Data sharing is not applicable to this article as no datasets were generated or analysed during the current study. The sequencing data that support the findings of this study will be made available on ClinVar under the following accession number: SCV002106410.

## Abbreviations

NS: Noonan syndrome
PV: Pathogenic variant
AD: Autosomal dominant
AR: Autosomal recessive
MRI: Magnetic resonance imaging
MRA: Magnetic resonance angiography
NF1: Neurofibromatosis type 1
NF2: Neurofibromatosis type 2
CALMs: Café-au-lait macules
SNP: Single nucleotide polymorphism
CNV: Copy number variant
NGS: Next generation sequencing
